# Supplementary material for: Metastable brain waves
Source: Nat Commun. 2019 Mar 5;10:1056. doi: 10.1038/s41467-019-08999-0 (PMC6401142; doi:10.1038/s41467-019-08999-0)
Supplement: Supplementary file 1 — Supplementary Information [file 41467_2019_8999_MOESM1_ESM.pdf]

Supplementary Information for:

Metastable brain waves

Roberts et al.

Supplementary Text

Coherence cross-correlation partitions

It turns out there is specific information in the interhemispheric partition (Supplementary Figure 3): front-back and top-bottom partitions are more similar to each other than they are to the left-right partition, possibly reflecting the unique 'gating role' of the corpus callosum. Thus some transitions occur across multiple partitions, while other partitions are sensitive to transitions with specific spatial (or topological) structure. This suggests a hierarchy of transitions: transitions for which the choice of spatial (or topological) partition matters, and more dramatic transitions that are evident in all partitions. Future work is required to catalog these possibilities.

Robustness across connectomes

We verified that the existence of wave patterns reproduces across connectomic data (Supplementary Movie 9). Waves similar to those in our fully-connected weighted connectome exist also in sparser networks (thresholded down to 10% density), and occur whether using traditional weight-based thresholding[1] or consistency-based thresholding[2] (mean nodal mean speeds 27 m s^{-1} and 29 m s^{-1}, respectively). Waves exist also on a 10%-density binary network thresholded by weight (with all weights set to 1; mean nodal mean speed 33 m s^{-1}). This is consistent with the lattice-like formation of binary networks, such that shorter connections are much more probable than longer connections. This is a natural substrate to support the increased local synchrony required for wave patterns. We also observed waves arising on two entirely independent connectomes: an elderly connectome derived using probabilistic tractography[3], and the 998-node Hagmann et al. connectome derived using deterministic tractography from diffusion spectrum imaging[4]. Notably, waves arise on the connectomes from individual subjects, with similar speeds (subject mean±SD = 33±2 m s^{-1}) and similar dwell times (subject mean±SD = 92±8 ms) across the cohort (Supplementary Figure 5a,b). These distributions are fairly narrow, but there is some variability due to the individual-subject connectomes. Future work will be needed to determine how the details of the patterns vary across subjects as well as the impact on wave properties of connectomic disturbances in brain disorders.

Robustness to initial conditions

We also verified robustness to initial conditions. Using an ensemble of 100 random initial conditions and the group average connectome with $c = 0.6, \tau = 1$ ms, we observed waves with little variability across the ensemble in the nodal mean speeds (ensemble mean±SD = 35.3±0.3 m s^{-1}) and dwell times (ensemble mean±SD = 89±2 ms) (Supplementary Figure 5c,d). Thus the choice of initial conditions only makes a small contribution to the summary statistics of these waves.

Robustness to noise

We verified that wave patterns persist in the presence of weak noise (Supplementary Movie 10). These wave patterns have lower local synchrony than the noise-free case but also exhibit metastability (Supplementary Figure 6). Interestingly, weak noise also increases the global synchrony and dwell times, similar to the effects of stochastic resonance in other systems[5]. Further increases in noise decrease local synchrony and extinguish the waves (at fixed coupling).

Robustness to choice of model

We verified that emergent waves are not restricted to our particular choice of neural mass model. We tested two additional models: a network extension of the Wilson-Cowan model[6-8] and the Kuramoto model[9,10]; see Methods for details. In both models we found large-scale waves (Supplementary Movie 11). In the Wilson-Cowan case, we observed complex dynamics with smooth waves in competition with partially-coherent activity that slowly intrudes onto the waves, leaving wave-like but less-coherent dynamics in its wake. This coexistence of coherent and incoherent states is an example of a chimera state[11]. In the Kuramoto case we observed waves when the model is in a regime of partial synchronization. Both of these models have complex dependencies on parameter values, similar to the neural mass model studied here.

Supplementary references

1 Rubinov, M. & Sporns, O. Complex network measures of brain connectivity: uses and interpretations. *Neuroimage* **52**, 1059-1069 (2010).

2 Roberts, J. A., Perry, A., Roberts, G., Mitchell, P. B. & Breakspear, M. Consistency-based thresholding of the human connectome. *Neuroimage* **145**, 118-129, doi:10.1016/j.neuroimage.2016.09.053 (2017).

3 Perry, A. *et al.* The Organisation of the Elderly Connectome. *Neuroimage* **114**, 414-426 (2015).

4 Hagmann, P. *et al.* Mapping the structural core of human cerebral cortex. *PLoS Biol.* **6**, e159 (2008).

5 Collins, J. J., Chow, C. C. & Imhoff, T. T. Stochastic resonance without tuning. *Nature* **376**, 236 (1995).

6 Hlinka, J. & Coombes, S. Using computational models to relate structural and functional brain connectivity. *Eur. J. Neurosci.* **36**, 2137-2145 (2012).

7 Wilson, H. R. & Cowan, J. D. Excitatory and inhibitory interactions in localized populations of model neurons. *Biophys. J.* **12**, 1-24 (1972).

8 Abeysuriya, R. G. *et al.* A biophysical model of dynamic balancing of excitation and inhibition in fast oscillatory large-scale networks. *PLoS Comput. Biol.* **14**, e1006007 (2018).

9 Gollo, L. L., Roberts, J. A. & Cocchi, L. Mapping how local perturbations influence systems-level brain dynamics. *Neuroimage* **160**, 97-112 (2017).

10 Breakspear, M., Heitmann, S. & Daffertshofer, A. Generative models of cortical oscillations: neurobiological implications of the Kuramoto model. *Front. Hum. Neurosci.* **4**, 190 (2010).
11 Laing, C. R. The dynamics of chimera states in heterogeneous Kuramoto networks. *Physica D: Nonlinear Phenomena* **238**, 1569-1588 (2009).

Supplementary Figures

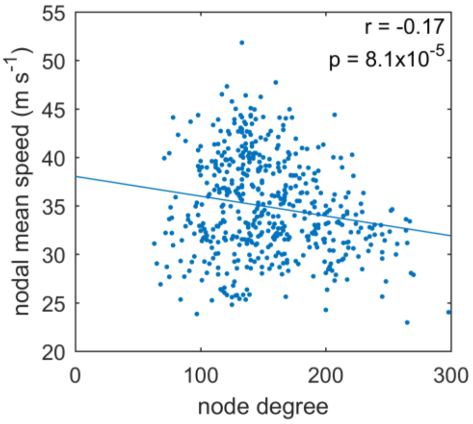

Supplementary Figure 1: Correlation between nodal mean speed and node degree. Degrees are calculated for the network thresholded to 30% density. Line is a least-squares fit. Source data are provided as a Source Data file.

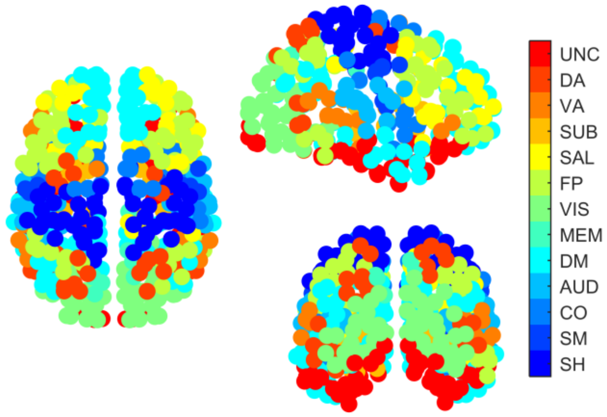

Supplementary Figure 2: Spatial map of functional networks. Nodes are labeled by color according to their functional network membership: SH=Somatomotor Hand, SM=Somatomotor Mouth, CO=Cingulo-Opercular, AUD=Auditory, DM=Default Mode, MEM=Memory, VIS=Visual, FP=Fronto-Parietal, SAL=Salience, SUB=Subcortical, VA=Ventral Attention, DA=Dorsal Attention, UNC=Unclassified.

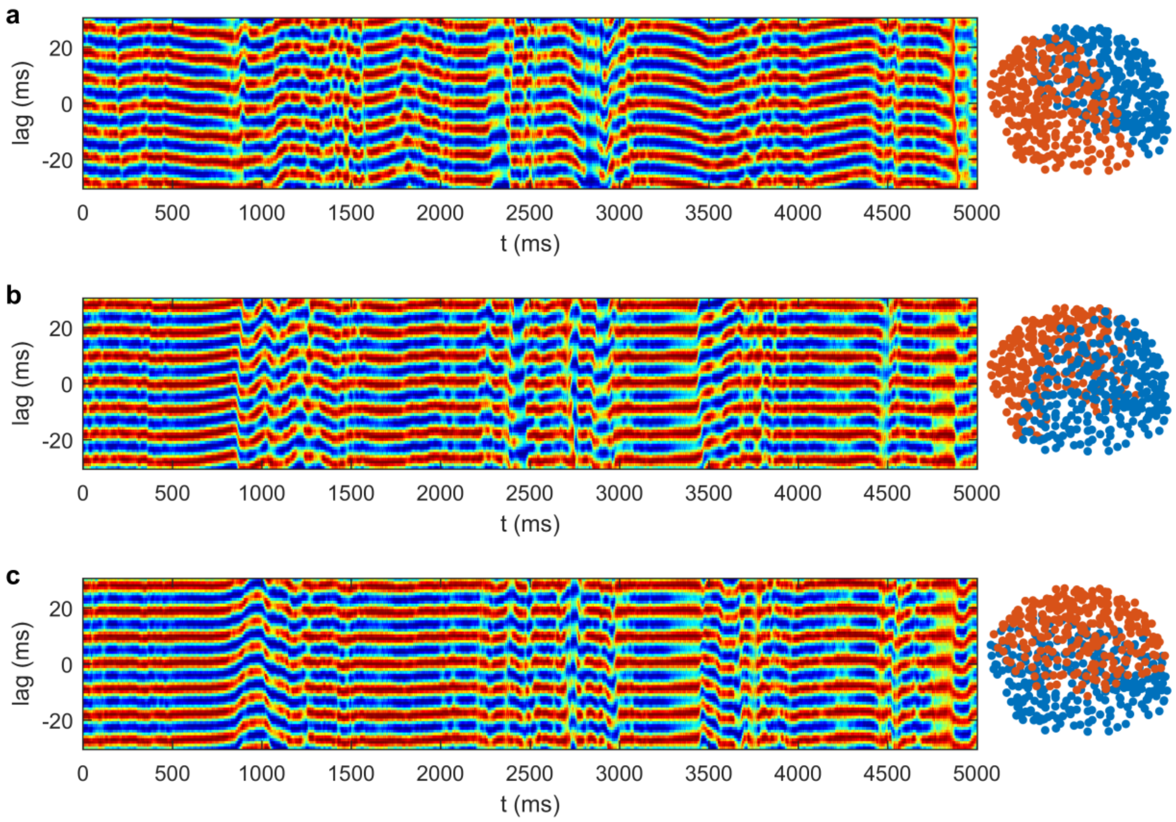

Supplementary Figure 3: Coherence cross-correlations for different partitions of the brain. (**a**) Left-right interhemispheric cross-correlation. (**b**) Anteroposterior cross-correlation. (**c**) Dorsoventral cross-correlation. Sliding-window cross-correlations are calculated on the same time series in all three panels. Node colors (right column) denote the partitions formed by median split in each direction.

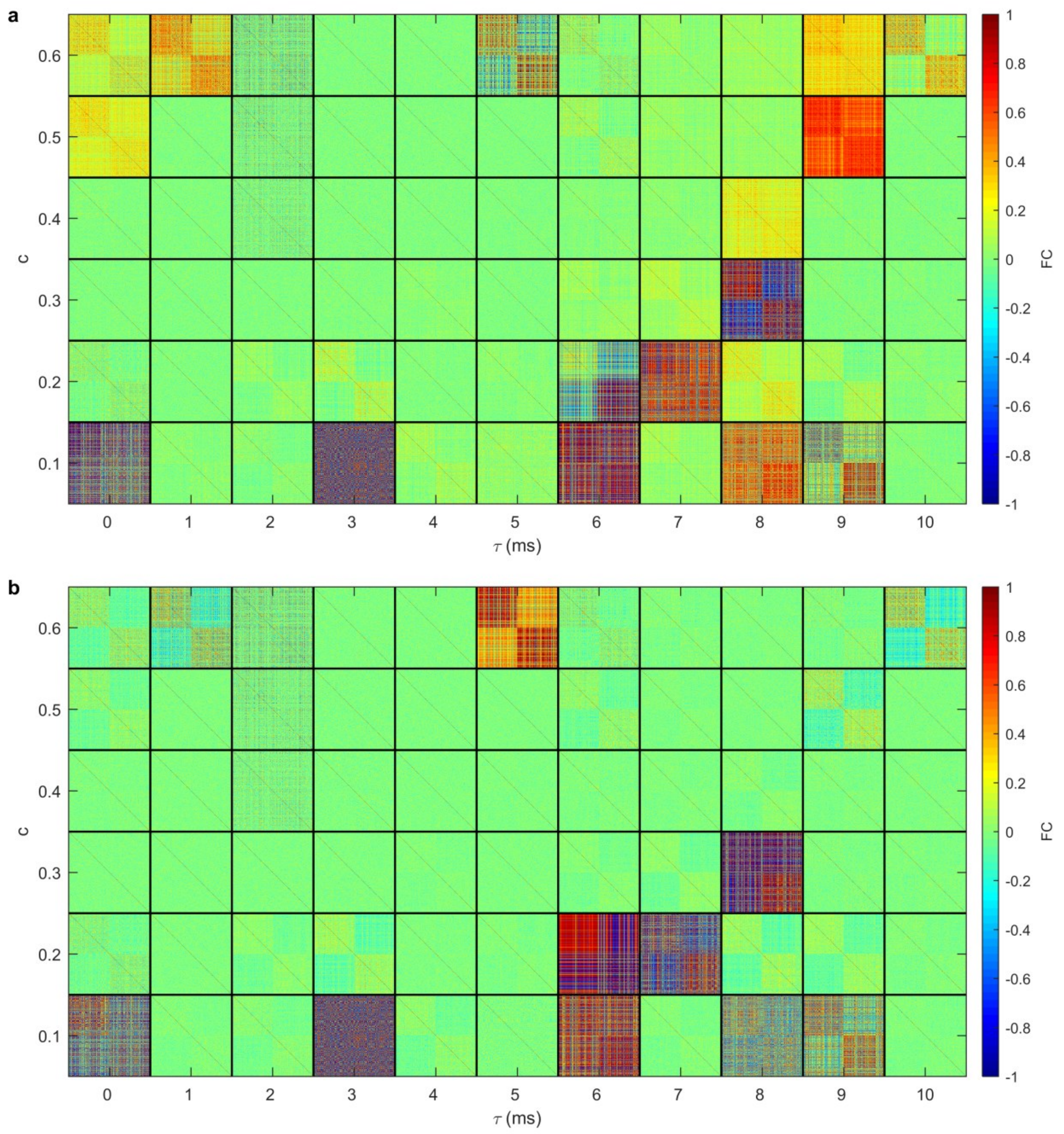

Supplementary Figure 4: FC matrices calculated on model BOLD time series. **(a)** BOLD FC without global signal regression. **(b)** BOLD FC with global signal regression.

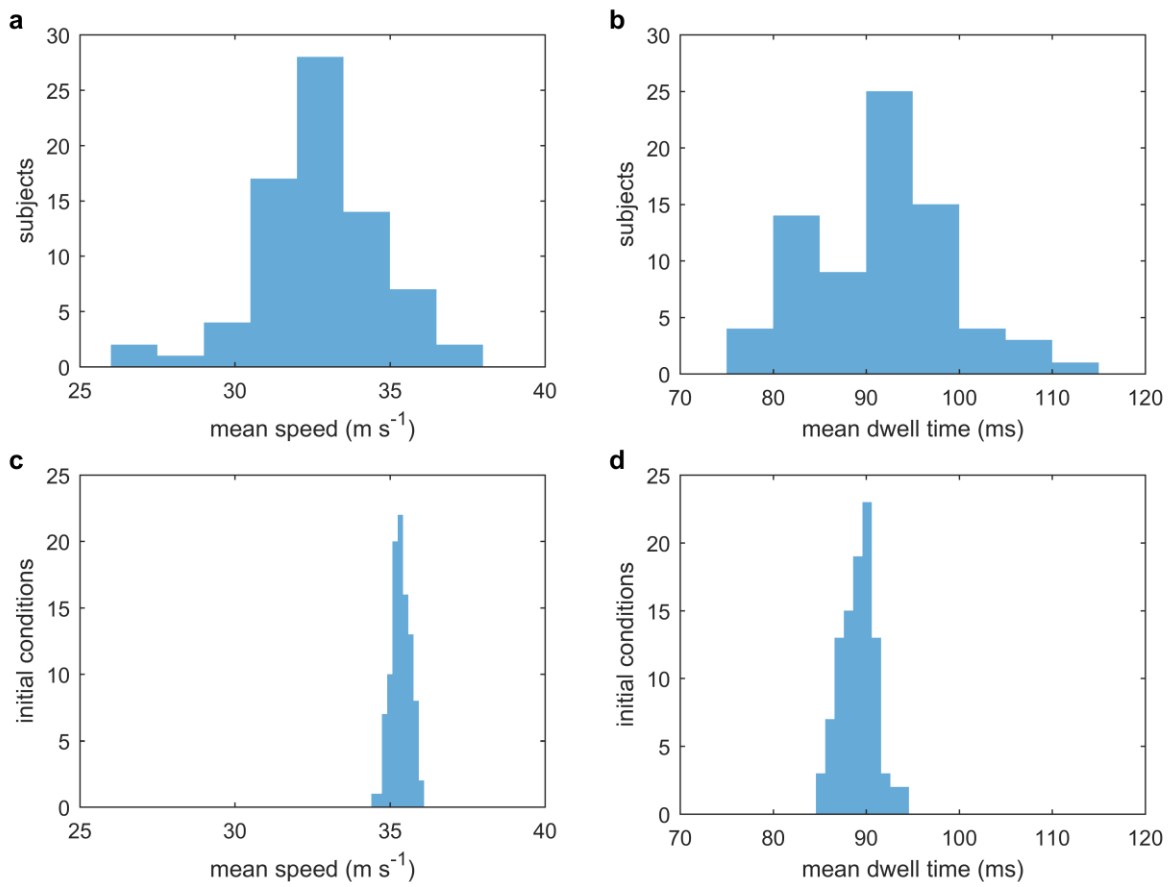

Supplementary Figure 5: Wave summary statistics across subjects (top row) and initial conditions (bottom row). Simulations performed for c=0.6, $\tau = 1$ ms. (**a**) Histogram across 75 subjects of mean speeds across all regions and times. (**b**) Histogram across subjects of mean dwell times. (**c**) Histogram across an ensemble of 100 random initial conditions (for the group mean connectome) of mean speeds across all regions and times. (**d**) Histogram across initial conditions of mean dwell times. Source data are provided as a Source Data file.

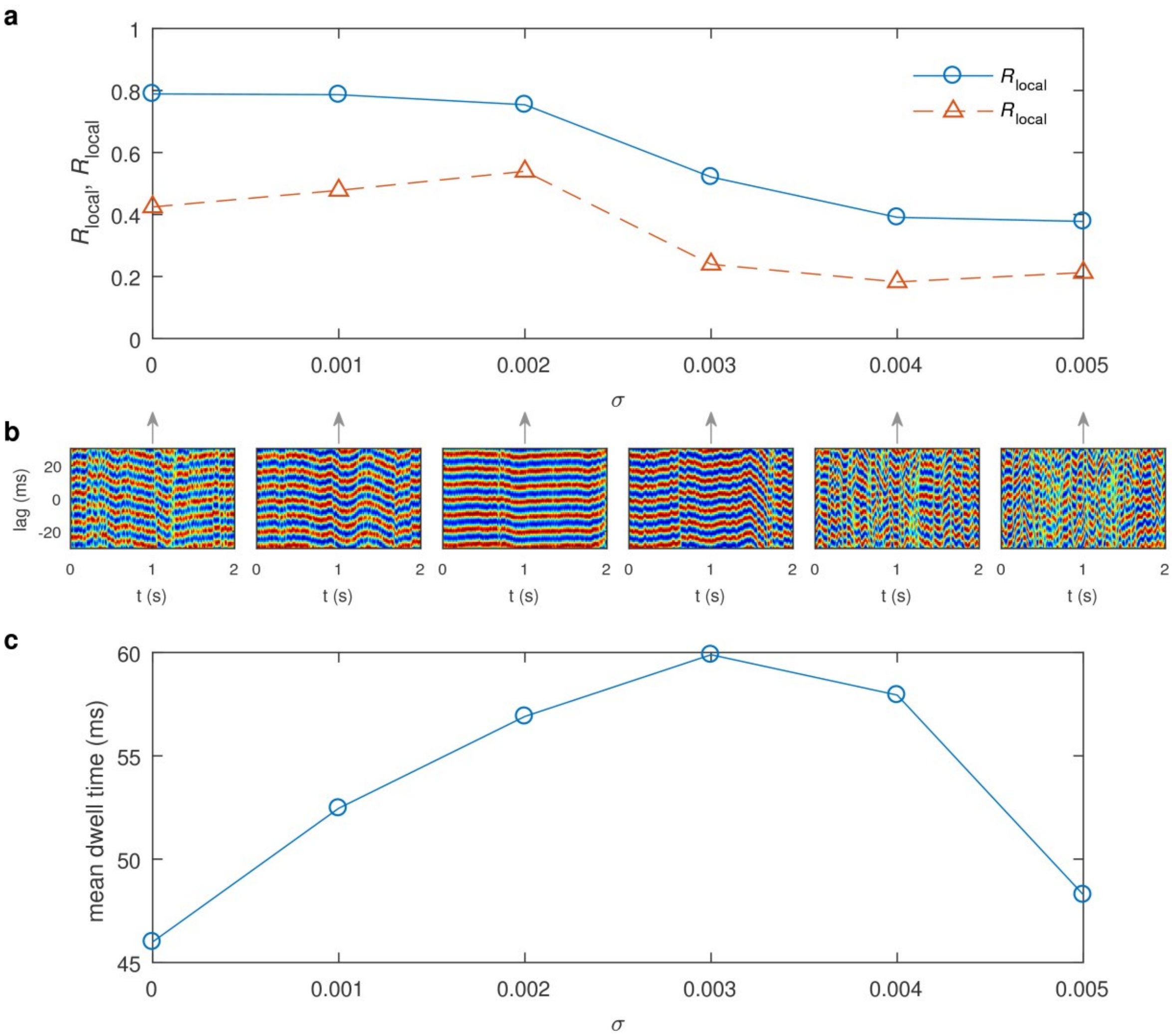

Supplementary Figure 6: Metastable transitions exist in the presence of noise. (**a**) Local and global synchrony as a function of additive noise amplitude σ. Lines show synchronization order parameters R_{local} (solid) and R_{global} (dashed). (**b**) Interhemispheric cross-correlation functions for the six values of σ indicated by arrows. (**c**) Mean dwell time as a function of σ. Note the slight decrease in local synchrony, increase in global synchrony and longer dwell times for weak noise, $\sigma \leq 0.002$. Simulations performed for $\tau = 0, c = 0.6$. Source data are provided as a Source Data file.

Supplementary Tables

Supplementary Table 1

Statistics for comparisons in Fig. 7c,d, bolded entries correspond to the colored comparisons in the figure. Numbers are two-tailed *p*-values for all pair-wise t-tests between the numbers of visits to each functional network, corrected for multiple comparisons (Bonferroni). Upper triangle is for comparisons between sink visits and functional networks, lower triangle (shaded) is for comparisons between source visits and functional networks.

	SH	SM	CO	AUD	DM	MEM	VIS	FP	SAL	SUB	VA	DA	UNC
SH		1	0.46	1	1	1	1	1	1	0.43	1	1	1
SM	1		0.97	1	1	1	1	1	1	0.81	1	1	1
CO	1	1		0.053	**0.039**	1	**0.033**	0.57	1	1	**0.0048**	1	**0.0033**
AUD	0.25	1	1		1	1	1	1	1	0.051	1	1	1
DM	**0.0053**	1	1	1		1	1	1	1	**0.048**	1	1	1
MEM	1	1	1	1	1		1	1	1	1	1	1	1
VIS	**0.0034**	1	1	1	1	0.8		1	1	**0.038**	1	1	1
FP	1	1	1	1	1	1	1		1	0.54	1	1	1
SAL	0.81	1	1	1	1	1	1	1		1	0.47	1	1
SUB	**0.0068**	1	1	1	1	0.39	1	1	1		**0.0049**	1	**0.0045**
VA	0.11	1	1	1	1	1	1	1	1	1		1	1
DA	0.82	1	1	1	1	1	1	1	1	1	1		1
UNC	**7.8×10^{-6}**	1	0.36	1	1	0.1	1	0.088	1	1	1	1	

Supplementary Table 2

Statistics for comparisons in Fig. 7e,f, bolded entries correspond to the colored comparisons in the figure. Numbers are two-tailed *p*-values for all pair-wise t-tests between the numbers of visits to each category, corrected for multiple comparisons (Bonferroni). Upper triangle is for comparisons between sink visits and hub status, lower triangle (shaded) is for comparisons between source visits and hub status.

	Hubs	Feeders	Non-hubs
Hubs		**0.00016**	1
Feeders	0.25		**0.00024**
Non-hubs	**0.0093**	0.11	

Supplementary Table 3

Parameters for the neural mass model (in nondimensional units)

Parameter	Description	Value
g_{Ca}	Maximal conductance of calcium ion channel	1
g_{Na}	Maximal conductance of sodium ion channel	6.7
g_K	Maximal conductance of potassium ion channel	2
g_L	Conductance of passive leaky membrane	0.5
V_{Ca}	Equilibrium potential of calcium ion channel	1
V_{Na}	Equilibrium potential of sodium ion channel	0.53
V_K	Equilibrium potential of potassium ion channel	-0.7
V_L	Equilibrium potential of passive leaky membrane	-0.5
T_{Ca}	Threshold for calcium ion channels	-0.01
T_{Na}	Threshold for sodium ion channels	0.3
T_K	Threshold for potassium ion channels	0
δ_{Ca}	Threshold standard deviation for calcium ion channels	0.15
δ_{Na}	Threshold standard deviation for sodium ion channels	0.15
δ_K	Threshold standard deviation for potassium ion channels	0.3
V_T	Firing threshold potential for excitatory population	0
Z_T	Firing threshold potential for inhibitory population	0
δ_V	Firing threshold standard deviation for excitatory population	0.65
δ_Z	Firing threshold standard deviation for inhibitory population	0.65
$Q_{V\max}$	Maximum firing rate for excitatory population	1
$Q_{Z\max}$	Maximum firing rate for inhibitory population	1
b	Rate constant for inhibitory dynamics	0.1
ϕ	Rate constant for potassium dynamics	0.7
r_{NMDA}	Ratio of NMDA to AMPA receptors	0.25
a_{ee}	Synaptic strength from excitatory to excitatory	0.36
a_{ie}	Synaptic strength from inhibitory to excitatory	2
a_{ne}	Synaptic strength from nonspecific to excitatory	1
a_{ei}	Synaptic strength from excitatory to inhibitory	2
a_{ni}	Synaptic strength from nonspecific to inhibitory	0.4
I_0	Mean nonspecific input strength	0.3
