# Supplementary material for: Metastable brain waves
Source: Nat Commun. 2019 Mar 5;10:1056. doi: 10.1038/s41467-019-08999-0 (PMC6401142; doi:10.1038/s41467-019-08999-0)
Supplement: Supplementary file 3 — Description of Additional Supplementary Files [file 41467_2019_8999_MOESM3_ESM.pdf]

Description of Additional Supplementary Files

Supplementary Movie 1

Traveling wave. An example of a traveling wave pattern, for coupling $c = 0.6$ and delay $\tau = 1$ ms, as in Figure 1a.

Supplementary Movie 2

Rotating wave. An example of a rotating wave pattern, for coupling $c = 0.6$ and delay $\tau = 1$ ms, as in Figure 1b.

Supplementary Movie 3

Sources and sinks. An example of a wave pattern with sources and sinks, for coupling $c = 0.6$ and delay $\tau = 1$ ms, as in Figure 1c.

Supplementary Movie 4

Example of spontaneous metastable transitions. Top row shows three views of the brain (l-r: top, right, back). Bottom row shows the interhemispheric cross-correlation (see Fig. 3), with the time of the frame frames above annotated by the sliding vertical line.

Supplementary Movie 5

Waves, flow vectors, and streamlines. Left: Pyramidal membrane potential, same dynamics as in Supplementary Movie 4. Middle: Unit vectors for the direction of instantaneous flow. Color denotes angle in the plane. Right: Streamlines propagated forward (blue) and backward (red) from the seed positions. Highlighted clusters of points are sinks (asterisks) and sources (circles), colored by position along the dorsoventral axis (red=high, blue=low).

Supplementary Movie 6

Streamlines and reconfigurations of sources and sinks. Streamlines propagated forward (blue) and backward (red) from the seed positions. Highlighted clusters of points are sinks (asterisks) and sources (circles), colored by position along the dorsoventral axis (red=high, blue=low). Three views: top (left), right side (middle), and back (right).

Supplementary Movie 7

Mosaic of brain dynamics across (c, τ)-space. Rows from bottom to top denote c=0.1,…,0.6 , columns from left to right denote τ=0, …, 10 ms. Each panel has color scale spanning the individual top and bottom percentile of pyramidal membrane potential.

Supplementary Movie 8

Examples of different dynamical regimes. Left to right: partially-coherent waves; weakly-coherent waves; lurching waves; periodic clusters; and aperiodic clusters.

Supplementary Movie 9

Examples of waves on different connectomes. Left to right: same connectome as main paper but at 10% density after thresholding by consistency; same connectome as main paper but at 10% density after thresholding by weight; same connectome as main paper but binarized at 10% density after thresholding by weight; a connectome from a healthy elderly population; the 998-node Hagmann et al. connectome.

Supplementary Movie 10

Examples of waves in the presence of weak additive noise inputs. Parameters c=0.6, τ=0. Left to right: No noise (σ=0), additive noise with σ=0.001, 0.002, 0.003, 0.004, 0.005.

Supplementary Movie 11

Examples of waves in different models. In all cases the connectivity is the same as in the main paper. Left to right: the Kuramoto model; the Wilson-Cowan model, showing a chimera state from early in the simulation run; the Wilson-Cowan model, from later in the same simulation run as the preceding frames, showing partially-coherent waves.
