# Supplementary material for: Metastable brain waves
Source: Nat Commun. 2019 Mar 5;10:1056. doi: 10.1038/s41467-019-08999-0 (PMC6401142; doi:10.1038/s41467-019-08999-0)
Supplement: Supplementary file 15 — Reporting Summary [file 41467_2019_8999_MOESM15_ESM.pdf]

natureresearch

Corresponding author(s): Roberts

Last updated by author(s): Jan 31, 2019

Reporting Summary

Nature Research wishes to improve the reproducibility of the work that we publish. This form provides structure for consistency and transparency in reporting. For further information on Nature Research policies, see Authors & Referees and the Editorial Policy Checklist.

Statistics

For all statistical analyses, confirm that the following items are present in the figure legend, table legend, main text, or Methods section.

n/a	Confirmed	
☐	☒	The exact sample size (n) for each experimental group/condition, given as a discrete number and unit of measurement
☐	☒	A statement on whether measurements were taken from distinct samples or whether the same sample was measured repeatedly
☐	☒	The statistical test(s) used AND whether they are one- or two-sided<br>*Only common tests should be described solely by name; describe more complex techniques in the Methods section.*
☐	☒	A description of all covariates tested
☐	☒	A description of any assumptions or corrections, such as tests of normality and adjustment for multiple comparisons
☐	☒	A full description of the statistical parameters including central tendency (e.g. means) or other basic estimates (e.g. regression coefficient) AND variation (e.g. standard deviation) or associated estimates of uncertainty (e.g. confidence intervals)
☐	☒	For null hypothesis testing, the test statistic (e.g. F, t, r) with confidence intervals, effect sizes, degrees of freedom and P value noted<br>*Give P values as exact values whenever suitable.*
☒	☐	For Bayesian analysis, information on the choice of priors and Markov chain Monte Carlo settings
☒	☐	For hierarchical and complex designs, identification of the appropriate level for tests and full reporting of outcomes
☐	☒	Estimates of effect sizes (e.g. Cohen's d, Pearson's r), indicating how they were calculated

Our web collection on statistics for biologists contains articles on many of the points above.

Software and code

Policy information about availability of computer code

Data collection	Preprocessing of all diffusion-weighted (DW) images was performed using FSL (Smith et al. 2004).<br>All tractography data were then analyzed using the freely available MRtrix software (Tournier et al., 2012).<br>Preprocessing of functional imaging data was performed using SPM8 in MATLAB 2012b.
Data analysis	Matlab 2017a.<br>Code is available at www.sng.org.au/Downloads.

For manuscripts utilizing custom algorithms or software that are central to the research but not yet described in published literature, software must be made available to editors/reviewers. We strongly encourage code deposition in a community repository (e.g. GitHub). See the Nature Research guidelines for submitting code & software for further information.

Data

Policy information about availability of data

All manuscripts must include a data availability statement. This statement should provide the following information, where applicable:

- Accession codes, unique identifiers, or web links for publicly available datasets
- A list of figures that have associated raw data
- A description of any restrictions on data availability

All data are available from the corresponding authors upon request. The source data underlying Figures 2b-d, 4, 7, and 10 and Supplementary Figures 1, 5, and 6a,c are provided as a Source Data file.

Field-specific reporting

Please select the one below that is the best fit for your research. If you are not sure, read the appropriate sections before making your selection.

☒ Life sciences ☐ Behavioural & social sciences ☐ Ecological, evolutionary & environmental sciences

For a reference copy of the document with all sections, see nature.com/documents/nr-reporting-summary-flat.pdf

Life sciences study design

All studies must disclose on these points even when the disclosure is negative.

Sample size

This is predominantly a computational modeling study, using diffusion imaging data from a cohort of 75 healthy young adults (aged 17-30) to determine the coupling parameters of a brain network model and compare the model dynamics to functional imaging data from the same subjects (69 participants had both the diffusion and functional data). Modeled functional connectivity and state lifetimes were also compared to a empirical estimates from resting-state MEG data from a cohort of 55 healthy participants. These were existing and previously published data sets. These sample sizes are sufficient to detect modest effect sizes as per Roberts et al. (2016) and Vidaurre et al. (2018) that previously used these datasets. Due to the discovery nature of the present modeling study, no explicit prior power calculations were possible.

Data exclusions

No data from these previously published studies were excluded from the present study.

Replication

We replicated our principal findings across a range of (i) coupling strengths and delays; (ii) connectomes, including different processing steps (threshold methods and densities) and different datasets (individuals vs the mean connectivity, an elderly connectome, and a widely-used diffusion spectrum imaging dataset); (iii) initial conditions in the simulations; and (iv) different models (Wilson-Cowan, Kuramoto).
To ensure that our findings can be reliably reproduced, our code is available online (www.sng.org.au/Downloads).
The connectivity data will be available upon request.

Randomization

This is not a randomized treatment study. Participants in the studies from where we sourced our data were recruited by advertisement. All participants meeting specified exclusion and inclusion criteria were recruited into the study.

Blinding

Blinding is not relevant to this study because it is not a randomized treatment study.

Reporting for specific materials, systems and methods

We require information from authors about some types of materials, experimental systems and methods used in many studies. Here, indicate whether each material, system or method listed is relevant to your study. If you are not sure if a list item applies to your research, read the appropriate section before selecting a response.

Materials & experimental systems

n/a	Involved in the study	
☒	☐	Antibodies
☒	☐	Eukaryotic cell lines
☒	☐	Palaeontology
☒	☐	Animals and other organisms
☐	☒	Human research participants
☒	☐	Clinical data

Methods

n/a	Involved in the study	
☒	☐	ChIP-seq
☒	☐	Flow cytometry
☐	☒	MRI-based neuroimaging

Human research participants

Policy information about studies involving human research participants

Population characteristics

We used existing data to build a model and test its predictions from two independent data sets - a normative cohort of 75 healthy young adults (aged 17-30, 47 females) for the structural and functional connectomes; and a normative cohort of 55 healthy participants (aged 18-48, 20 females) for the MEG lifetimes and functional connectivity. These were existing and previously published data sets.

Recruitment

Participants were recruited by advertisement. All participants meeting specified exclusion and inclusion criteria were recruited into the study.

Ethics oversight

This study was approved by the QIMR Berghofer Human Research Ethics Committee.

Note that full information on the approval of the study protocol must also be provided in the manuscript.

Magnetic resonance imaging

Experimental design

Design type	Computational modeling of brain dynamics. The model uses as its connectivity parameters structural connectivity data from 75 healthy young adults (age 17-30). The emergent dynamics are compared to resting-state functional imaging from the 69 subjects who had this data available.
Design specifications	Resting-state functional imaging was acquired for 6 minutes. All MRI data were acquired according to standard protocols and as described in the Methods section.
Behavioral performance measures	There are no behavioral tasks in this study.

Acquisition

Imaging type(s)	Diffusion MRI and functional MRI.
Field strength	3T.
Sequence & imaging parameters	Diffusion MRI data were acquired for the first data set using a Philips 3 T Achieva Quasar Dual MRI scanner (Philips Medical System, Best, The Netherlands) using a single-shot echo-planar imaging (EPI) sequence (TR = 7767 ms, TE = 68 ms).<br><br>Functional images were collected using a T2* weighted echo-planar imaging sequence (TE = 30 ms, TR = 2000 ms, flip angle = 90º, FOV 250 mm, 136 x 136 mm matrix size in Fourier space) and consisted of twenty-nine contiguous 4.5 mm axial slices (no gap) covering the entire brain.
Area of acquisition	Whole brain.

Diffusion MRI ☒ Used ☐ Not used

Parameters: For each diffusion scan, 32 gradient directions (b = 1000 s/mm2) and a non-diffusion-weighted acquisition (b = 0 s/mm2) were acquired over a 96 × 96 image matrix (field of view 240 mm × 240 mm × 137.5 mm), with a slice thickness of 2.5 mm and no gap, reconstructed to yield 1 mm × 1 mm × 2.5 mm voxels (where the longer dimension is along the dorsoventral axis). Two sets of diffusion scans were acquired for each subject.

Preprocessing

Preprocessing software	Preprocessing of all diffusion-weighted (DW) images was performed using FSL (Smith et al. 2004).<br>All tractography data were then analysed using the freely available MRtrix software (Tournier et al., 2012).<br>Preprocessing of functional imaging data was performed using SPM8 in MATLAB 2012b.
Normalization	The fMRI data used for the functional connectivity analyses were spatially normalized into standard Montreal Neurological Institute (MNI) space (MNI/CBM avg 152 T2* template) using a 12-parameter affine transformation implemented in SPM8.
Normalization template	To derive structural connectivity matrices from the diffusion MRI data, anatomical nodes were pre-defined by subdividing the standard AAL (Tzourio-Mazoyer et al., 2002) template into 513 cortical and sub-cortical regions of approximately uniform size (Zalesky et al., 2010). Parcellations within single-subject space were achieved by employing affine linear registrations within the FSL software package (Smith et al., 2004).
Noise and artifact removal	To correct for head motion, the gradient direction matrix was rotated using a custom algorithm available in MRtrix software (Tournier et al., 2012; Leemans and Jones, 2009; Raffelt et al., 2012). To reduce spatial intensity inhomogeneities, intensity normalisation was performed on the b0 image and subsequently applied to all diffusion-weighted (DW) images (Sled et al., 1998). Lastly, a Higher Order Model Outlier Rejection model (Pannek et al., 2012) identified voxels with residual outliers in the DWsignal.
Volume censoring	No volume censoring used.

Statistical modeling & inference

Model type and settings	(1) fMRI data were used to compare modeled to empirical functional connectivity, by performing a Pearson's correlation between all edges in the group-mean empirical functional connectivity and the corresponding edges in the model (where FC was simulated using a group-mean structural connectome).<br><br>(2) ANOVAs were used to determine whether model wave dynamics were heterogeneous with respect to structural hub status or functional network label (Power et al. 2011). Mass univariate t-tests (corrected for multiple comparisons) were then used to find which subsets of nodes drove the overall heterogeneity.
Effect(s) tested	(1) The effect is the correlation between modeled and empirical group-level functional connectivity.

(2) The effect is heterogeneity in the node-level numbers of sources/sinks with respect to hub status or functional network label.

Specify type of analysis: ☐ Whole brain ☐ ROI-based ☒ Both

Anatomical location(s): All analyses were performed at the whole brain level. There were no a priori region of interest analyses. We did use an atlas-based parcellation as described above for the purposes of defining structural connectivity between brain regions. However, we included all parcels from all gray matter regions in our analyses.

Statistic type for inference (See Eklund et al. 2016): We did not analyse any fMRI contrasts and therefore did not perform voxel- or cluster-wise family-wise correction (as addressed in Eklund).

Correction: Mass univariate t-tests were corrected for multiple comparisons using Bonferroni correction.

Models & analysis

n/a	Involved in the study
☐	☒ Functional and/or effective connectivity
☐	☒ Graph analysis
☐	☒ Multivariate modeling or predictive analysis

Functional and/or effective connectivity: Resting-state functional connectivity was calculated using Pearson correlations between each pair of regions.

Graph analysis: For an analysis of model dynamics (the numbers of source and sink visits to each region), the regions were partitioned according to the hub status of their structural connectivity by using the weighted node strength.

Multivariate modeling and predictive analysis: We used ANOVAs with independent variables of hub status (as above) or functional network label (Power et al. 2011) and dependent variables of number of source or sink visits to each region.
